# Striving for safety: communicating and deciding in sociotechnical systems

**DOI:** 10.1080/00140139.2015.1015621

**Published:** 2015-03-11

**Authors:** John M. Flach, John S. Carroll, Marvin J. Dainoff, W. Ian Hamilton

**Affiliations:** ^a^ Department of Psychology, Wright State University, 335 Fawcett Hall, Dayton, OH, USA; ^b^ Sloan School of Management, Massachusetts Institute of Technology, Cambridge, MA, USA; ^c^ Liberty Mutual Research Institute for Safety, Center for Behavioral Sciences, 71 Frankland Road, Hopkinton, MA, USA; ^d^ ERM, Ltd., 1 Castle Park, Bristol, UK

**Keywords:** safety, sociotechnical systems, communications, decision-making, observability, controllability, dynamical systems

## Abstract

How do communications and decisions impact the safety of sociotechnical systems? This paper frames this question in the context of a dynamic system of nested sub-systems. Communications are related to the construct of observability (i.e. how components integrate information to assess the state with respect to local and global constraints). Decisions are related to the construct of controllability (i.e. how component sub-systems act to meet local and global safety goals). The safety dynamics of sociotechnical systems are evaluated as a function of the coupling between observability and controllability across multiple closed-loop components. Two very different domains (nuclear power and the limited service food industry) provide examples to illustrate how this framework might be applied. While the dynamical systems framework does not offer simple prescriptions for achieving safety, it does provide guides for exploring specific systems to consider the potential fit between organisational structures and work demands, and for generalising across different systems regarding how safety can be managed.

**Practitioner Summary:** While offering no simple prescriptions about how to achieve safety in sociotechnical systems, this paper develops a theoretical framework based on dynamical systems theory as a practical guide for generalising from basic research to work domains and for generalising across alternative work domains to better understand how patterns of communication and decision-making impact system safety.

## 1. Introduction

The objective for this paper is to explore how communications and decisions impact the safety of complex sociotechnical systems. In order to ground these explorations, we focus on two case studies involving safety issues in two very different sociotechnical systems – the Millstone Nuclear Power Station and the fast (limited service) food industry. These two choices reflect two qualitatively different aspects of the safety problem. The Millstone case illustrates issues associated with process control, where the focus is on managing a complex, high-risk technology (i.e. a nuclear power plant). In contrast, the fast food industry case illustrates issues associated with workplace safety, where the focus is on managing the safety of individuals in the workplace (i.e. minimising accidents associated with slips and falls). By choosing two very distinct safety challenges, we hope to highlight both: (A) common challenges that must be addressed in the study of all sociotechnical systems and (B) important general properties of sociotechnical systems relevant to communicating and deciding. In particular, we will frame the problems of communicating and deciding relative to the dynamics of *observing* and *controlling* within a dynamic self-organising system. It is our hope that this framing of the problem will lead to more productive collaborations between researchers and practitioners and to greater convergence across the many disciplines that can offer potential insights into this complex problem of safety. For alternative general discussions of a control theoretic framework in relation to modelling the performance of human–machine systems, consider the following sources: Flach et al. (2011, 2013), Jagacinski and Flach (2003), Leveson (2012) and Sheridan and Ferrell (1974).

## 2. What is a sociotechnical system?

A significant challenge that researchers interested in safety in sociotechnical systems must face is to identify the ‘system’ to be studied. Over the last 50 years, there is an important message that emerges from research on human cognition and organisational sensemaking – *Context Matters!* Simon (1969) illustrated this with his famous analogy of the ant on the beach, where he suggested that it would be impossible to model the ant's behaviour without including properties of the beach in the model. In other words, the message is that cognitive systems are adaptive systems and, in order to understand the adaptive dynamics, it is important to include the ‘situations’ that the cognitive agents/organisations are adapting to as part of the ‘system’ being modelled. This insight has been emphasised by constructs such as ‘situated cognition’ (e.g. Suchman 1987; Hutchins 1995) and ‘ecological rationality’ (e.g. Todd and Gigerenzer 2012). Thus, a core principal of a sociotechnical systems approach is that the system of interest must include both the ‘social’ and ‘technical’ aspects of work as interdependent components of the work system (e.g. Clegg 2000).

**Figure 1:**
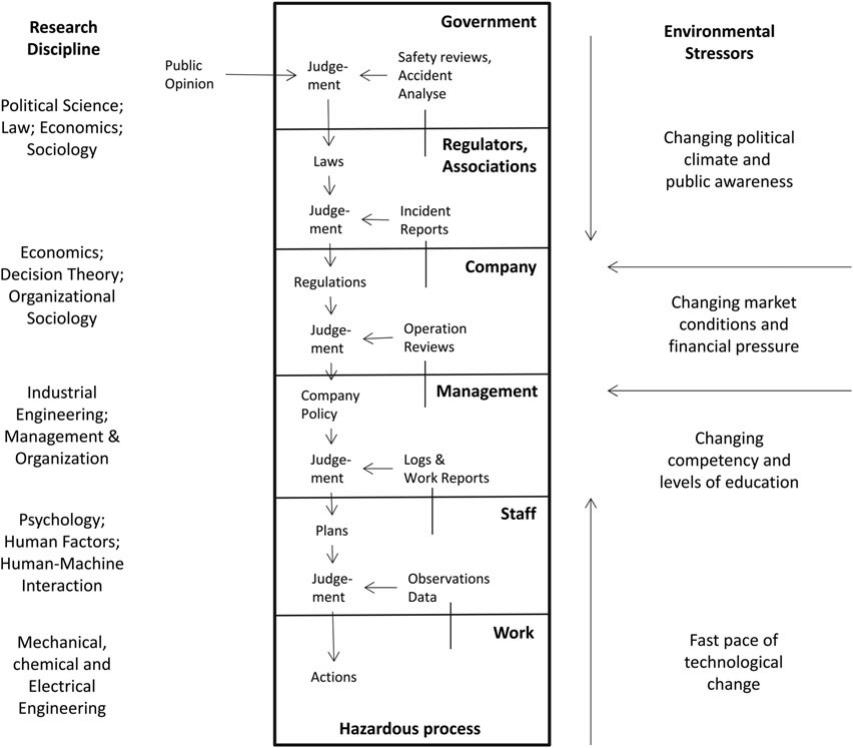
This figure adapted from Rasmussen and Svedung (2000) illustrates the multiple social layers that shape the behaviour of sociotechnical systems. Such couplings have important implication for the breadth of analysis and for the range of disciplines that must collaborate to fully understand these complex systems.

In the specific context of system safety, Rasmussen, Pejtersen, and Goodstein (1994) and Leveson (2012) emphasise the tight coupling between the technical system (e.g. a power plant or a fast food chain) and the larger social context (e.g. regulatory, political and cultural ecologies). For example, Rasmussen and Svedung (2000) argue that due to the fast pace of technological change, the scale of industrial installations, and the high degree of integration and coupling across systems, it is ‘becoming increasing difficult to model work organisations in isolation and to make small-scale, local experiments to evaluate models’ (10). Figure 1 (adapted from Rasmussen and Svedung) illustrates some of the ways that technical systems have become coupled to the larger social context. Figure 1 illustrates the multiple dimensions of the work organisation, the broad range of environmental stressors that shape the demands and opportunities, and the diverse range of disciplines that provide theoretical and empirical bases for addressing different properties of the sociotechnical system. Leveson (2012, 82, Figure 4.4.) provides an alternative, more detailed illustration of the couplings across multiple levels associated with the design and operation of a complex sociotechnical system. However, we will use the Rasmussen and Svedung model as a general framework for mapping out the details associated with our two cases.

### 2.1. Millstone

**Figure 2:**
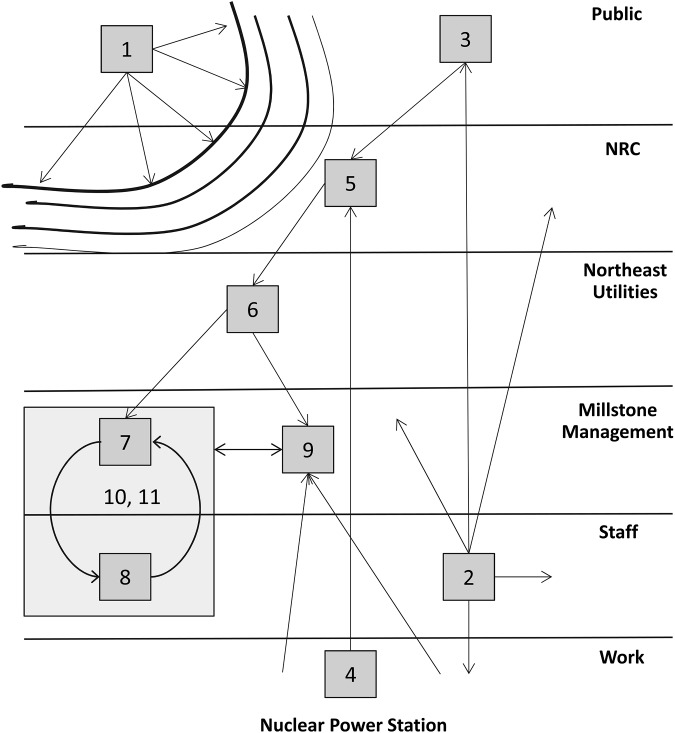
This figure adapted from Rasmussen and Svedung (2000) will be used to help illustrate the events associated with the struggle to manage safety at the Millstone Power Station.

Figure 2 represents some key events relative to the Millstone Nuclear Power Station's struggles to manage safety (Carroll and Hatkenaka 2001).^4^ The story of Millstone begins in the 1980s when a confluence of events set the stage for the emergence of serious safety concerns. These events included significant construction costs associated with the Unit 3 Reactor, the retirement of Millstone's pioneering CEO, and a change in the economic climate due to potential deregulation of energy production. The result was increased pressure to reduce costs in response to the economic context illustrated as *Block 1* in Figure 2. This increased economic pressure sent a ripple through the entire system that was felt at all levels. The ripples of these economic pressures were felt most acutely at the operational staff level where workers felt pressured to work closer to the safety margins and to delay enhancements that other plants in the industry were implementing. The result, represented by *Block 2*, was an impact on worker morale (e.g. dissatisfaction with management), on the performance of work (e.g. increasing backlogs in maintenance) and growing complaints to management, to regulatory agencies and to the public. For example, the U.S. Nuclear Regulatory Commission (NRC) was ‘receiving approximately 50 employee allegations annually’ (71).

Worker concerns about safety at Millstone eventually came to the attention of the broader public, and *Time* magazine began preparing a story about the ‘alleged harassment and intimidation of Millstone employees who raised safety concerns’ (71) [*Block 3*]. Knowledge that *Time* magazine was working on this story helped to stimulate the NRC to become more actively involved with the Millstone situation at the start of 1996. In January, Millstone was placed on the Watch List and, when each of the three reactors was shut down for refueling and various equipment problems [*Block 4*], ‘the NRC ordered all three to demonstrate compliance with their licenses, regulations and safety analyses before restarting’ (71) [*Block 5*]. In October, as the result of an NRC review, Northeast Nuclear Energy was directed to develop and implement a plan that would result in a ‘“safety-conscious work environment” in which employees could raise concerns without fear of retaliation – and management would take appropriate action’ (71). In addition, the NRC told Northeast Utilities to contract with an independent third party (subject to NRC approval) to oversee the development and implementation of the plan for addressing safety concerns.

In response to pressures from the NRC and its own internal assessment of the management problems at Millstone, Northeast Utilities hired a new leader, Bruce Kenyon, to oversee the nuclear programmes [*Block 6*]. Kenyon immediately began a dialogue with the working staff both to learn more about the problems and also to demonstrate his commitment to ‘do what was right’ (72) [*Blocks 7, 8, 10* and *11*]. *Block 7* represents the first step in establishing this dialogue, which involved a two-week assessment period including peer ratings of the management staff that reported to him, and concluded with the firing of two vice presidents and demotion of a third. Several months later, disciplinary actions taken against the manager of the oversight department for issues that had been around for many years had the unintended consequence that, in the context of the earlier work experiences [*Block 2*], was perceived by many employees as an attack on independent oversight [*Block 8*]. Thus, *Blocks 7* and *8* represent the initiation of a continuing closed-loop dialogue between workers and management. By ‘closed-loop’ we mean that communications from senders are two way: information, commands, requests and actions are interpreted by the audience which sends signals back to the senders in the form of more communications (including actions that speak louder than words, if anyone listens). Some additional key events in that dialogue included hiring a Navy admiral as the new vice president of oversight, who in turn hired his former chief of staff ‘to strengthen and run the employees concerns program’ (72) [*Block 10*]. Another important event was the establishment of a programme to train line management to work and communicate more effectively with employees to establish a safety conscious work environment [*Block 11*]. Also, the hiring of an independent, external oversight team [*Block 9*] played an important role in helping management to better understand the safety problems and in facilitating communications between workers and management.

It is impossible to represent the complete time history associated with the dialogue between management and staff [e.g. *Blocks 7, 8, 10* and *11*] without making Figure 2 too complex to be useful. The progress towards improved safety did not follow a simple, smooth, errorless path. It took time to rebuild the trust that had eroded between workers and management – well intended actions had unintended negative consequences (e.g. as a result of how they were perceived by workers) and some actions were simply wrong and required correction (e.g. two contractors who were fired after raising safety concerns were offered their jobs back). Thus, we have highlighted this as a closed-loop subsystem. The closed-loop dynamics of the dialogue between management and workers at Millstone will be examined in more detail in the context of the dynamics of communicating and deciding. The closed-loop nature of this dynamic has important implications for how we frame research on communications and decisions in sociotechnical systems.

### 2.2. Limited service food industry

**Figure 3:**
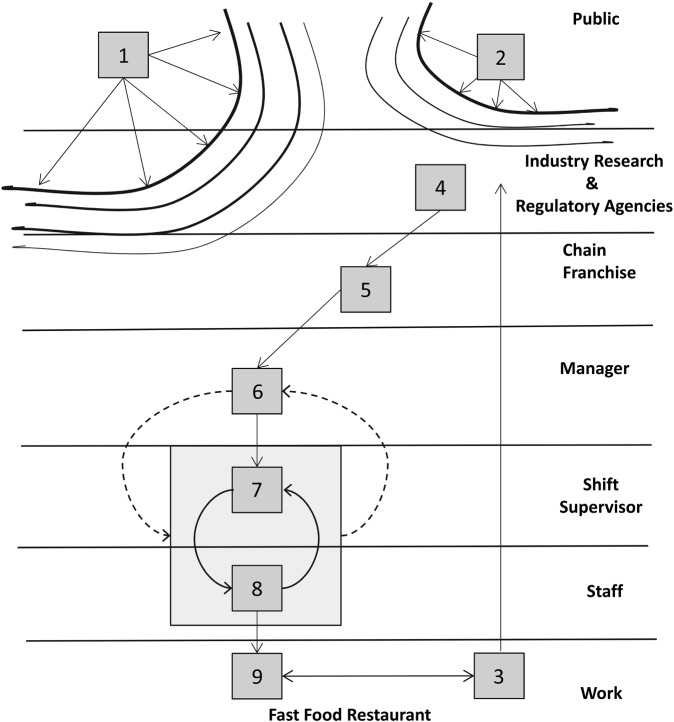
This figure adapted from Rasmussen and Svedung (2000) will be used to help illustrate the events associated with the struggle to manage slips and falls in the fast food industry.

Figure 3 provides a map of the sociotechnical landscape for some important events associated with slips and related injuries in the limited service or fast food industry (Verma et al. 2010, 2011).^5^ While falls are a function of multiple factors that include the design of the workplace and the nature of the work (Haslam and Stubbs 2006), this analysis focuses on a single industry (fast food restaurants) and a single factor (floor cleanliness). The focus of this story starts with *Block 3*, which represents slips in limited service restaurants. Slips and falls are the leading cause of injury in fast food restaurants. In the USA, they account for one-third of the disabling injuries. It has been estimated that the rate of falls over a two-year period is about 4.1 per 100 full-time-equivalent restaurant employees (Leamon and Murphy 1995). Other studies suggest that upwards of 60% of these falls can be attributed to slipping. In addition to falls, slips are also implicated in other accidents such as grease burns (Hayes-Lundy et al. 1991). Direct costs of slips, trips and fall-related injuries in the USA in 2010 were estimated to be $8.61 billion, according to the Liberty Mutual Workplace Safety Index (2012). This is a 37% increase from 1998, which is the greatest increase among major leading causes of workplace injury.

An important factor associated with slipping is the coefficient of friction (COF) for floors. For example, Verma et al. (2010) found that the rate of slipping decreased by 21% for each 0.1 increase in COF [*Block 4*]. However, the COF for surfaces will also be determined by whether they are clean. Many fast food franchises require that enzymatic floor cleaners be utilised in all their stores [*Block 5*]. In the Verma et al. studies, results indicated that 25 of the 36 restaurants studied (69%) used enzymatic floor cleaners that require cold water to be effective, because hot water deactivates the enzymes. *Block 2* represents a widespread assumption (or mental model) among the public that hotter water is more effective for cleaning than cooler water, and Verma et al. found that 62% of employees using enzymatic cleaners reported using warm or hot water when cleaning floors. By contrast, 98% of those using regular detergent-based cleaners appropriately used hot water. This mismatch may occur despite franchise mandates, training materials and guidance typically provided to store managers [*Block 5*], who, in turn, are responsible for selection and training of the supervisors and staff [*Block 6*] and making them aware of the cold-water requirements associated with the enzymatic cleaners. In reality, control may be much more diffuse and less hierarchical.

Shift supervisors [*Block 7*] directly supervise the staff [*Block 8*], who actually do the work of mopping the floors [*Block 9*], which in turn impacts the state of the floors and therefore the likelihood of slips [*Block 3*]. The kitchen area floors are mopped at least once a day (typically during the night shift) and usually more often. This is important, because managers will generally not be present during the night shift. Thus, it will often be up to the shift supervisors to monitor whether cold water is used with the enzymatic cleaners. Managers may not be in a position to observe slips if and when they occur due to other responsibilities and shift duration. Supervisors will probably only be aware of slips that result in injury or disruption of the normal workflow. Slips in the Verma et al. study were operationally defined in terms of weekly reports to the study team by individual participants. They were characterised as ‘slips’ and ‘major slips’, the latter resulting in fall or injury. The exact process of what is reported to whom within specific restaurants seems to vary. Thus, the dashed feedback to management reflects uncertainty about how much information relative to both slips and floor cleaning activities is being reported back to managers. On the other hand, the responsibility to see that supervisors and staff have the appropriate training and that they are following appropriate procedures with regard to floor cleaning falls with the managers.

A final factor that provides an important context for the interactions among managers, supervisors and staff is the economic constraints [*Block 1*] that keep the staff salaries in fast food restaurants near minimum wage. Restaurants also employ a large percentage of part time employees (students, care-givers, etc.). Hence, there are potentially high levels of staff and supervisor turnover. In addition, many stores operate with longer hours and multiple shifts, raising the issue of situational awareness transmission between shift supervisors and work crews from shift to shift. This interacts with the general public's assumptions about the need for hot water [*Block 2*] to make worker training and supervision a continual concern. This high turnover rate will have important implications for the effectiveness of communications and decisions with respect to countering incorrect assumptions among the general public and preventing slips due to improper cleaning practices. Verma et al. recommended a combination of administrative and engineering controls including a dispensing station with a cold-water-only supply/control valve.

### 2.3. A sociotechnical systems perspective

The main purpose of this first section was to reinforce and emphasise a ‘Sociotechnical SYSTEMS’ approach to safety, with a very strong emphasis on the term ‘systems’. As Leveson (2012) writes:

The systems' approach focuses on systems taken as a whole, not on the parts. It assumes that some properties of systems can be treated adequately only in their entirety, taking into account all facets relating the social to the technical aspects. These system properties derive from the relationships between the parts of systems: how the parts interact and fit together. (63)

In particular, a systems perspective on safety starts with the premise that ‘safety’ is an emergent property of the organised complexity. This means that any attempt to understand or to improve system safety based on reductionist assumptions (e.g. an exclusive focus on eliminating specific human error or on increasing component software or hardware reliability) will have limited effectiveness at best, and at worst may lead to naive models and interventions that can have potentially catastrophic unintended negative consequences. Each of the two cases introduced here illustrate the necessity of considering communications and decisions in the context of the larger social system within which they are embedded.

In the case of the Millstone Power Station, it seems evident that the economic climate was a factor contributing to the erosion of the safety culture and that the larger public (i.e. the attention of *Time* magazine) played a very important role in helping both the NRC and Northeast Utilities to recognise that there was a safety problem and to motivate them to initiate actions to make improvements. Furthermore, common industry patterns around directive management and mistrust between managers and workers made communication more difficult. In addition, in connecting the dots between different actions (e.g. replacing top managers) and the ultimate safety consequences of those actions, it becomes necessary to consider couplings that involve multiple levels (e.g. the trust of workers and the education of middle management relative to the values of top level management, the regulatory demands and the economic pressures).

Similarly, in the case of slip-related injuries in the fast food industry, it seems quite obvious that effective interventions will have to be framed in the context of the economic environment that contributes to high worker turnover rates and the cultural environment that creates expectations about cleaning effectiveness that are violated by the enzymatic cleaning solutions. Thus, rational decisions about the choice of a cleaning detergent at the franchise level can have unintended negative consequences for worker safety.

Now that the sociotechnical systems perspective has been established, the next section will specifically consider the implications for understanding and shaping communications and decision-making with the goal of improving safety. In particular, communicating and deciding will be explored in the context of a set of interacting, nested closed-loop dynamics that span the multiple social layers illustrated in Figure 1.

## 3. Observing and controlling: a closed-loop perspective on communicating and deciding

**Figure 4:**
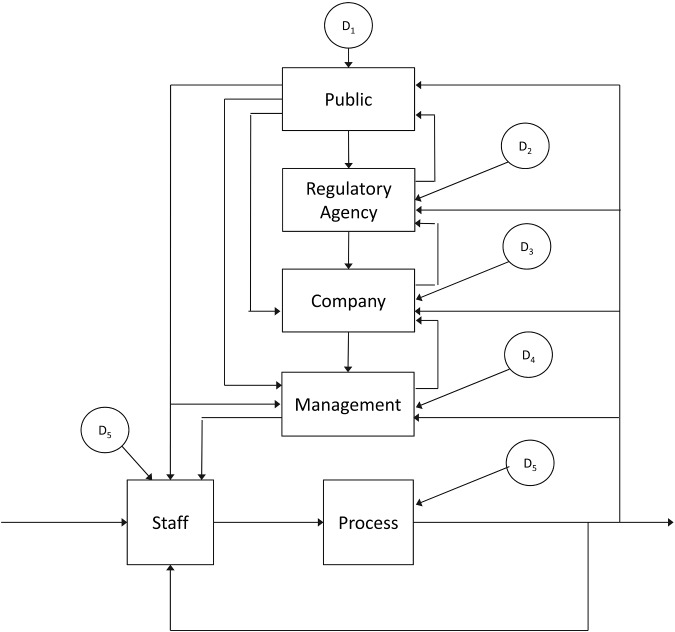
This figure is intended to illustrate some of the nested loops shaping performance in a complex sociotechnical system. Note that all possible loops are not depicted and that each of the components may have unique disturbances that influence its output.

Figure 4 provides an alternative representation that illustrates the coupling across levels in a sociotechnical system as a nesting of closed-loop couplings. Although only a subset of the various couplings is represented in the figure, in principle, every element in this system can be influenced by output from every other element and all elements may be affected by independent disturbances. The first point that we want to make with regard to this representation is that the arrows represent the flow of both ‘communications’ and ‘decisions’. In fact, almost any activity in this system will have a dual role, both as a decision/action (e.g. firing a vice president; or choosing a detergent) and as a communication (e.g. reflected in the perceptions of staff about management safety values; or about the proper way to clean floors). In essence, all decisions are communications and most communications have potential impact on decisions and actions. In this context, the common distinction between communications and decisions breaks down, and attention shifts to the coupling between communications (i.e. perceptions) and decisions (i.e. actions) relative to stability in a multi-layered adaptive control system. As an alternative, we suggest that a distinction that becomes more relevant to the dynamics of the coupled system is the distinction between observing/observability and controlling/controllability, where observability focuses on the ability to monitor the states of the system (e.g. in relation to constructs such as organisational sensemaking, recognition primed decisions, situation awareness and common ground); and controllability focuses on the ability to influence the system or move it from one state to another (e.g. leadership, authority and responsibility within the organisation). In complex organisations, observability will depend on effective communications and the effectiveness of decision-making can only be assessed relative to the constraints associated with controllability.

**Figure 5:**
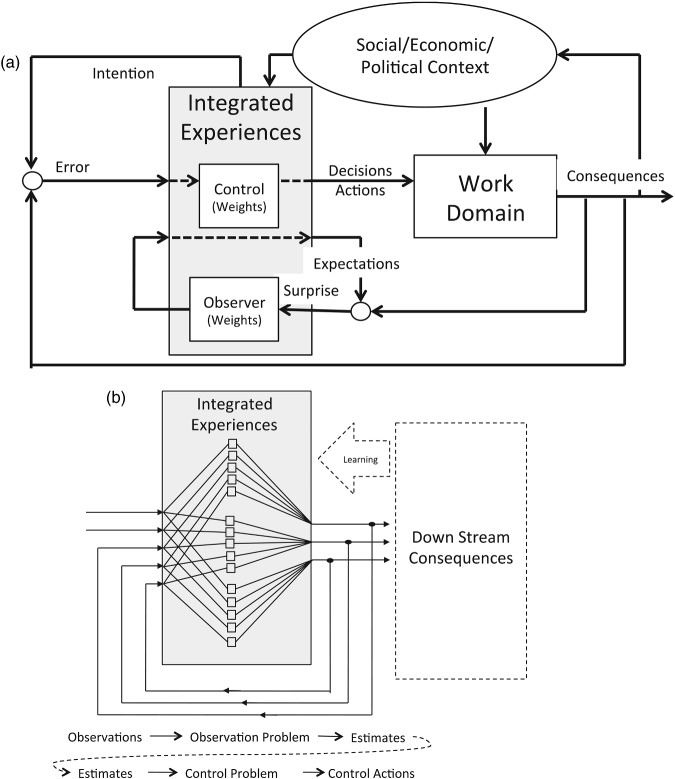
These figures are designed to help readers to visualise the general functional relations associated with observing and controlling: (A) illustrates the observer loop as nested within a control loop which is in turn nested within a learning loop; (B) illustrates the multi-dimensional nature of observation (reflecting the need to integrate multiple sources of information) and control (reflecting the need to coordinate multiple degrees of freedom). [See Flach et al. (2011) for a similar discussion in the context of driving safety].

Figure 5 provides two alternative visualisations to aid in our discussions of the observability and controllability problems within each of the component control systems within the larger sociotechnical system. Figure 5(A) emphasises the functional relations within each component loop within the larger system, and Figure 5(B) emphasises the multi-dimensional nature of the information flows associated with observing and controlling within each loop. The centre of Figure 5(A) is labelled ‘integrated experiences’ – this is the heart of the sensemaking process. The term ‘integrated’ is used explicitly to emphasise that the outputs of this system (i.e. decisions, actions and expectations) are dependent on an extended history of past experiences. In the argot of cognitive psychology, this element might represent the ‘schema’ or ‘mental model’ that is guiding expectations and actions and that is simultaneously being shaped by the consequent experiences. As Leveson (2012, 87) notes, ‘any controller – human or automated – needs a model of the process being controlled to control it effectively’. However, this model is not stationary: it reflects learning (the integration of experience) over time. Also, it is important to note that the work itself (i.e. the process being controlled or problem being solved) is also changing over time. Thus, a changing internal model is a necessary component in an adaptive control system.

The integrated experiences element is a component in three nested feedback loops. The inner-most loop represents the observation process with respect to direct work activities (e.g. operating a nuclear power plant or washing floors). In this loop, expectations are compared to the outputs of the controlled process and the differences (i.e. surprise) are potentially fed back as inputs to the integrated experiences. The primary component of this inner loop reflects the sensitivity to (or the weight/gain, emphasis associated with or attentiveness to) the feedback. This will determine the impact of a surprise at any specific time, relative to integrated prior experiences, in shaping the assessment of the situation. This loop might reflect the Millstone operators' assessment of the state of the nuclear processes that they are operating; or in the case of the fast food industry, this might reflect a worker's assessment of the requirements for cleaning floors.

The next inner-most loop represents the control loop, where outputs of the controlled process are compared to intentions, and the differences (i.e. errors) are fed back as inputs to the integrated experiences. The block element in this loop represents the sensitivity to deviations from intentions. In simple control systems, this component (e.g. the control gain) would determine the rate of responses to errors. However, more generally this might be analogous to the priority associated with actions towards satisfying the system objectives. Thus, this loop might reflect the decisions that operators at Millstone might make with regard to both controlling and maintaining the nuclear processes. In the case of the fast food industry, this loop might reflect the decisions of workers about what temperature of water to use to clean the floors.

Note that in simple control systems, the stability of the overall system will depend on the joint impact of both the control and observer components (e.g. gains). If the joint gain is too high, the system will ‘overreact’ and can actually make problems worse. If the joint gain is too low, then the system will be very slow and conservative in responding to problems. In a dynamic world where there are finite windows of opportunity to solve problems, inaction or responding too slow can also lead to instability. Thus, the gain elements reflect an important balancing act with respect to observability (differentiating signal from noise) and with respect to controllability (essentially a speed/accuracy tradeoff).

The outer-most loop represents the input to a component control system from the other levels in the sociotechnical system. In this loop, experiences are shaped by the feedback that is filtered through the larger socio-economic-political context. This loop will typically be integrating over longer time periods than the two inner loops, and the input from these levels can be best visualised as ‘constraints’ that shape the intentions (values and goals) and limit the degrees of freedom for selecting control solutions at the component level. These constraints can be thought of as a ‘mental model’ that might include the goals, values and alternatives that shape how information is integrated both for assessing the state of the system and for selecting the appropriate actions. Ideally, in an adaptive system (e.g. a learning organisation), the feedback from this loop will reflect learning from successes and failures over the history of the organisation that might facilitate increasing ‘expertise’ with respect to a particular component control problem (i.e. smarter or more stable control solutions). In the Millstone case, this might reflect input to the operators from management and regulatory agencies with respect to the appropriate balance between efficiency and safety. Note that the messages may not be consistent and may vary over time. In the fast food industry case, this might reflect the instruction and guidance that workers receive from their management, as well as experiences in their everyday lives with respect to proper cleaning procedures (e.g. if enzymatic cleaners become more widely used, then widespread assumptions about water temperature might change).

Although the signals in typical control diagrams such as Figure 5(A) are represented as single arrows, in sociotechnical systems these signals will typically reflect multiple sources of information with respect to multiple dimensions (i.e. states) of the control problem. Figure 5(B) is included to emphasise that in complex sociotechnical systems, both the observation and control processes will involve multiple dimensions of feedback and multiple dimensions (i.e. possibilities or degrees of freedom) for action. In Figure 5(B) the transfer function (e.g. the internal model) of the integrated experience component is represented as a network of weights (as opposed to a single gain as in Figure 5(A)) – where each box represents a differential weighting function for each of many inputs relative to each of the many outputs. This representation is akin to Brunswik's (1956) Lens Model for perception. Note that the multiple signal lines do not represent nested control loops, but rather a single control loop that is multi-dimensional (i.e. includes multiple sources of feedback and multiple action possibilities). However, we have embedded this in a second outer loop to reflect the potential for the weights in the ‘lens’ to be adjusted as a consequence of the internal model that is shaped by the coupling with other levels in the sociotechnical system. The wide arrow (labelled ‘learning’) reflects a process whereby the relative weights in the lens (i.e. the functional relationships or transfer function) are adjusted as a result of the couplings across levels in the sociotechnical system. In essence, the weights in the lens component of this system are one way to operationalise a changing ‘internal model’ (i.e. a mental model or implicit theory of action) that translates observations into expectations and/or that translates errors into action. Note that the lines in Figure 5(B) represent signals that are operated on by the functional relationships within the box (i.e. the weights in the lens) and translated into action. However, the wide learning arrow represents an operation on the functional relationships (i.e. changing the weights in the lens). Thus, the impact of learning from the broader experience associated with cross-level coupling within the larger system is to tune (or reshape) the dynamics of the multi-dimensional perception action loop. As noted earlier, the learning loop will be operating at a different (slower) time constant than the inner loop. Thus, for example, operators at Millstone may be receiving changing messages with respect to the appropriate emphasis on safety from management that will impact how they might approach maintenance of the system (e.g. employees who see management ignoring safety concerns may stop reporting the concerns or may voice those concerns through a different channel – complaining to regulatory agencies). In the case of the fast food industry, expectations based on a long history of using non-enzymatic cleaners will shape how workers might interpret the feedback with respect to slips in the workplace. For example, whether they attribute the slip to the cleanliness of the floors or to other factors and whether they respond by changing how they clean the floors.

As we explore the constructs of observability and controllability in the context of complex sociotechnical systems, it is important to keep both Figures 4 and 5 in mind. Every loop in Figure 4 represents distinct observer and controller constraints. Thus, every line in Figure 4 represents multiple inputs and every box in Figure 4 contains its own ‘lenses’ for mapping these multiple inputs into expectations and actions. In addition, information that is essential to observability and controllability for one loop may be noise with respect to another loop. Thus, we are dealing with a network of observers and controllers that have to satisfy local stability constraints and also must coordinate to satisfy more global stability constraints. However, the general principles of observability and controllability can be applied both locally and globally.

Thus, at a global organisational level we can talk about observability and controllability (e.g. organisational sensemaking and learning). These will depend on coordination across many local closed-loop dynamics at levels associated with units within the organisation and with individuals within the units. We can also consider local dynamics of observability and controllability (e.g. individual situation awareness and expertise). Thus, not only is there a need for balance within each loop, but there is also a potential need to balance stability demands within a loop against the demands of other loops in order to achieve a globally stable solution. For example, in the case of Millstone, the resources available for addressing safety concerns at the operator level will be constrained by decisions at another level with respect to meeting the demands for economic viability. In the case of the fast food industry, the potential benefits of choosing an enzymatic-based cleaner at one level must be assessed relative to the associated demands for training and supervision at another level.

In simple linear control systems, it is possible to separate the observer and controller functions into independent components that serially determine the stability of the sociotechnical system (e.g. see Pew and Baron 1978). Thus, the ‘estimates’ of output by the observer become the inputs to the controller. However, in complex, nonlinear systems, the observer and controller functions will typically be inextricably coupled, jointly contributing to the integrated experiences that in turn shape actions and expectations. Thus, it becomes impossible to disambiguate or disentangle the individual contributions to the system dynamics (i.e. as separate gains). For example, new leaders in the Millstone plant must simultaneously solve the safety problems, while they are still learning or discovering what the problems are. Thus, early actions may not have the intended consequences (with respect to the control problem), and the associated surprises will be potential information for adjusting the internal model of the state of the system (the observer problem).

In Figure 5(B), the diagram can represent either the observer or the controller as isolated components in the forward loop; or it can represent the joint impact of both (i.e. the coordinated coupling of perception and action). In the second case, the weights at the nodes represent the internal model that coordinates expectations and actions. For pedagogical reasons we will begin by considering the observer and controller functions independently and then in a final section we will consider the joint implications for stability and safety. Ultimately, the stability of a control system will be a joint function of observability and controllability. Thus, we feel it is essential to approach them as complementary features of a single system, rather than as subsystems that can be understood in isolation.

### 3.1. Observing

For social scientists, a good context for thinking about the observing problem is the theory of signal detectability. In essence, the problem is to infer the actual state of the system based on noisy observations. In particular, the problem is determining whether any particular observation (e.g. a safety problem or a slip) is simply associated with the natural complexity of the work domain, or whether that observation is symptomatic of a systemic problem that might require action. For example, in the case of Millstone, one might consider management's problem of determining whether the safety complaints of the staff represented actual threats to the overall safety of the plant – or perhaps simply the complaints of unsatisfied or overly cautious workers who are not aware of the overall economic context. Would an investment in an employee concerns programme simply be ‘a legal defense fund for bad employees?’ Or at another level consider the staff's problem of determining whether management actions represented a genuine increased interest in improving plant safety or just a ploy to placate workers and regulators. Was the management initiative to strengthen the employee concerns programme simply a ‘look good and feel good’ gimmick? In the case of the fast food industry, consider the problem of a shift supervisor trying to determine whether a specific fall was due to the work conditions or to careless actions of an accident-prone employee. There will be evidence to support multiple attributions.

The key point, with respect to the signal detectability problem, is that solving the observer problem is not about finding an absolute truth. Rather, it is a problem of balancing one source of error against another. Reducing misses (increasing hits) comes at the cost of increasing false alarms. Thus, in the case of Millstone, responding immediately to make a change as a result of every concern voiced by employees would potentially result in much wasted money and effort. In this context, the observer problem becomes one of finding the right decision criteria for judging which complaints to act on and which complaints to ignore. In more colloquial terms, the question is to find the right level of trust. Too much trust results in wasted time and effort. Too little trust results in an erosion of safety leading to potential catastrophe. The key is to calibrate trust relative to the properties of signal and noise (the degree of uncertainty) and to the relative payoffs associated with the potential consequences, which themselves depend on context or history. In an environment of mistrust, erring on the side of extra investment is probably wise; over time, as trust is developed, the effective level of trust changes (just as would be the case with any resource).

The ‘gain/bias’ component in the inner loop of Figure 5(A) and the weights in Figure 5(B) reflect the calibration of the observer process. These components reflect the weights given to multiple sources of information relative to the expectations based on prior experience. In simple observer systems this might be implemented as a gain or weight that determines how much the estimate of the state of the system will be changed as a function of the current level of surprise. If the gain is high, then the system will dramatically change its expectation when there is a surprise. If the gain is low, then the system will tend to be conservative in changing its expectations. If gain is too high, the system will become unstable as it ‘chases’ the noise. If gain is too low, then the system will be sluggish in responding to real changes in the state of the work domain.

An important implication of taking the signal detection perspective on the problem is that a satisfactory or optimal solution cannot be specified without taking the larger context (e.g. the economic consequences) into account. In other words, the social-economic-political context matters! Thus, in order to approach the observer problem with respect to determining satisfactory solutions (e.g. to choose an appropriate level of gain), it is necessary to take into account the value systems (i.e. payoff matrix or cost function). In essence, the observer problem requires a sociotechnical systems perspective.

While the signal detection problem illustrates the need to balance one source of error against the other, the full complexity of the observer problem requires considering the dynamics of sampling over time and the potential that the situation is changing. In the Millstone case, the problem is not about responding to individual staff observations about particular safety issues, but rather integrating across many observations over months, years and even decades. The Millstone plant had an excellent safety record and was noted for engineering excellence through the 1970s and 1980s. However, due to the changing economic environment (and perhaps due to growing complacency), the state of the organisation was changing (i.e. the safety culture was eroding and the expectations of external parties were rising), while the state of the industry was also changing with new technology and regulatory requirements, thus creating a gap of expectations among some stakeholders. The observer problem involves sampling over time to detect this gradual change. The expectation based on the history of operations through the 1970s and 1980s was that the Millstone systems were well managed and safe – however, that belief was no longer justified by the mid-1990s. However, this change could not be detected in any single observation – it involves detecting patterns over time.

In the case of slipping in the fast food industry, the magnitude of the problem is impossible to detect at the level of individual restaurants. In fact, Figure 3 suggests that the feedback loop with regard to the industry-wide slip problem is only closed at the level of large-scale research projects conducted by scientists such as Verma et al. The number of slips reported or resulting in an injury experienced in a particular restaurant will be low (few samples) and the magnitude of the noise (i.e. in terms of competing attributions) is immense. It is very easy to attribute any single slip to a multitude of factors such as individual carelessness. In this system, as in other systems (most notably healthcare) where accidents (errors) are local and incidents are distributed over many different units, it can be very difficult to detect a systemic problem. However, when the data are integrated across many units, the magnitude of the problem becomes more apparent. This illustrates one of the important roles for regulatory agencies and industry groups (e.g. The Institute of Nuclear Power Operations) in the observer process – to integrate across large samples in order to detect significant safety issues that are hidden in the noise at more local levels. This is currently occurring at the injury level in the U.S. Occupational Safety and Health Administration. It is interesting to note that the problem of the enzymatic cleaners was a serendipitous discovery in a research programme that was evaluating COFs relative to the cost/benefits of providing workers with slip-resistant shoes.

In taking into account the dynamics associated with sampling and changing system states, it should be apparent that the capacity to sample, and thus the ability to differentiate signal from noise, will be different at each of the different layers in the sociotechnical system. The implication is that a satisfactory assessment of the state of a sociotechnical system is not possible without coordinating information across the various levels. No particular level at either Millstone or in the fast food industry has privileged access to understanding the complete state of the system. For example, while detecting the full magnitude of the slipping safety issue may require levels of integration that are possible only at higher levels in the sociotechnical system, improving the situation will typically depend on properties of lower level loops, such as the ability of managers to ensure that supervisors and staff are trained to use the detergents properly.

Another aspect of the observer problem that is not well represented in the signal detection paradigm (that is generally framed in the context of perception) is the need to actively explore in order to access information. A classic example in the control literature involves the problem of a driver detecting potential changes in driving dynamics (e.g. a change in road surfaces such as black ice, or a change in the braking dynamics due to wet brakes). In order to detect the changing dynamics, it will sometimes be necessary to act (e.g. jiggle the steering wheel, or tap the brakes) with the goal of creating feedback to ‘test’ for changes in the state of the system. In the Millstone report (Carroll and Hatkenaka 2001), this is reflected in the discussion of ‘learning by doing’. With regard to the observation problem, the new management was ‘surprised’ by the reactions of workers to firing contractors and disciplining managers. This surprise is an indication of a mismatch between the expectations of management and the actual state of the system. The feedback from these actions was very important for building an accurate assessment of the state of the organisation – and for achieving an appropriate level of trust between management and staff. This illustrates a truism for effective observing – it is necessary to learn from mistakes, thus you have to make mistakes first in order to learn. Jiggling your steering wheel to test for black ice will be perceived as error with respect to the narrow goal of steering the car (i.e. controlling), but it may provide essential information with regard to the higher goal of driving safely.

We feel that there is a large degree of consensus in the literature about the significance of the observing problem in complex work environments. However, this consensus can be masked by the diverse jargon that has developed around this problem (e.g. situation awareness, recognition-primed decision-making, organisational sensemaking and common ground). For example, the terms ‘situation awareness’ and ‘recognition-primed decision-making’ have emerged from research comparing individual novice and expert performance in complex environments. This research shows that much of the difference can be attributed to the ability of experts to accurately assess or ‘recognise’ the state of the situation. This literature suggests that the differential quality of meeting the observer demands of complexity accounts for much of the variance between individual novices and experts, rather than differential abilities to ‘choose/decide’ or differential action capabilities. Individual experts are typically more accurate in assessing the state of the situation, and this assessment then translates into more effective action. The observer metaphor can provide a means for making generalisations from the literature on individual expertise to the problem of organisational sensemaking.

The term ‘common ground’ reflects the need for collaboration across many levels in an organisation in order to successfully accomplish complex work. This term arises in the communication literature and is viewed as an important pre-requisite for effective conversations (Clark and Brennan 1991). It has also been used in the organisational performance literature as a prerequisite for effective coordination (e.g. Convertino et al. 2011). As we noted earlier, the observation problem must be addressed in each of the many different control loops in an organisation. In terms of Figure 5(B), each loop will have its own internal model (i.e. weights for integrating information through the lens), reflecting its own learning history. Thus, it can often be the case that the same objective event is perceived differently due to the different learning histories at different levels of the organisation. Or, the intention motivating a message at one level may be completely misunderstood by others who are interpreting the intention through a different lens. It seems fairly obvious that an important contributing factor to the erosion of safety in the Millstone plants was the disappearing common ground between management and staff. This probably resulted from changes in management (e.g. retirement of the founding CEO) and the significant impact of the economic forces (e.g. impending deregulation) on the lens through which the new management was evaluating the state of the plants.

Common ground also figures significantly in the slipping problem in the fast food industry. The reality of enzymatic cleaners (i.e. require cold water to be effective) and the popular assumptions that many staff workers hold (i.e. hotter water is more effective) represent a clear disconnect between worker perceptions and the demands of the work. Thus, for example, guidance from management that clean floors are important for reducing slips might be perceived by workers as a requirement to use hotter water. A typical solution to this mismatch would be a training intervention directed to recalibrate the workers' expectations (i.e. train them to use cold water with enzymatic cleaners). However, because of the high turnover rate at the staff level, this training will be a continuing problem, unless the popular assumptions change due to increasing availability and general use of enzymatic cleaners. Thus, it may be important to at least consider alternative changes that reduce the potential conflict with general public beliefs. Suggestions include removing hot water sources, automating the mixing process so that workers do not control the water temperature, or utilise alternative detergents that are not enzymatic based.

In sum, to the extent that it is true that ‘perception is reality’, the observer problem reflects the difficulty of coordinating among many potentially different realities reflecting the differential experiences of the many individuals and groups who must cooperate to achieve good results for the sociotechnical system. It seems obvious that in both cases, a contributing factor to the safety problems was breakdowns in communications across levels (problems with observability). For example, within the fast food industry we wonder whether franchises appreciate the implications of their choice of an enzymatic cleaner on the slip rates. We wonder whether managers know about the requirement to use cold water with enzymatic cleaners, whether they know about what temperature water their employees are using, whether they are informed about specific slip incidents or whether they are fully aware of the extent of the safety issue. We wonder whether shift supervisors know about the requirements for cold water or the significance of the slip risks for the industry. We also wonder whether they know to report slip incidents to managers. The overall impression we get from the recent studies of slips in the fast food industry is that information feedback is lacking, resulting in poor situation awareness throughout the system with regard to the slip problem. Again, this was a serendipitous discovery that surprised the researchers whose interest was the potential impact of non-slip shoes on safety.

### 3.2. Controlling

As reflected in Figure 5, the control problem shares many properties with the observer problem. Whereas the observer problem is to integrate over experiences to make an accurate assessment of the situation, the control problem is to integrate over experiences to choose the right actions to satisfy the system goals or to minimise errors. Both problems involve integrating information from diverse sources over extended time histories, and both problems are inherently closed-loop. Finally, both problems involve an inherent tradeoff associated with the ultimate sensitivity to feedback.

As with the observer problem, the effectiveness of simple control systems often depends on sensitivity to error (i.e. the loop gain). If the gain is too high, then the system will tend to overcorrect errors and in the extreme the system will become unstable. In the aviation domain, high gain is associated with the problem of ‘pilot-induced oscillations’, where the over-corrections of the pilot lead to unstable control. If the gain is too low, then the control system will be sluggish or slow to correct errors. In finding the correct gain, a major constraint will be the time constraints associated with the control loop (e.g. lags and windows of opportunity associated with the effects of an action and delays associated with feedback about the impact of the actions). In general, when there are long delays between action and effect or feedback relative to the effect, then the range of stable gains will be very narrow, i.e. the system will be hard to control. In these cases, actions should be small and tentative in order to minimise the risk of instability.

As with the observer, the optimal solution can only be determined relative to an external value system (i.e. cost function). Typically, value functions reflect the costs of both error and effort, as well as the anticipated benefits associated with the positive consequences of action. An optimal solution will minimise costs and maximise benefits, but given that multiple stakeholders experience costs and benefits differently, there will be necessary tradeoffs both within component control loops and across the loops at different levels in the larger sociotechnical system. In the context of Millstone, one example of a control problem was the challenge of responding to staff concerns in order to both improve safety and improve the level of trust or confidence of the operators. Note that a good solution to the control problem is not simply to respond to every concern. It requires that management find an effective balance between the costs of action, the threats associated with the concerns and other potential consequences such as the public perception and the political and economic implications of those perceptions.

Like the observer problem, the control problem in sociotechnical systems typically involves many interconnected loops spanning different levels within the system. In motor control, this is referred to as the ‘degrees of freedom’ problem. One aspect of this problem is that actions intended to correct errors within one loop can be disturbances with respect to other loops. Thus, the net effect can be particularly unsatisfying. For example, bringing your head up too early when trying to ‘drive’ a golf ball can result in a very unsatisfying result. One of the challenges relative to solving the degrees of freedom problem is to ‘lock out’ a subset of control loops (e.g. fix your head position and lock the elbow of the leading arm) to minimise disturbances, so that control can be isolated to a few control loops that are particularly relevant to the goals. In sociotechnical systems, this has to do with authority and responsibility – which loops should be in control (determining the actions) and which loops should be ‘locked out’ from the action paths.

The Millstone case clearly illustrates how actions intended to correct errors in one loop (e.g. disciplining the manager of oversight) can result in disturbances (i.e. unintended negative consequences) with respect to other control loops (e.g. building trust with the staff). Similarly, the fast food industry case illustrates how a decision at one level intended to improve conditions (e.g. the choice of a more effective enzymatic cleaner) may have unintended negative impact on control loops at lower levels due to the choice of hot water by staff who are also intending to get maximal cleaning effectiveness. Both loops are acting to achieve the same goal, but because of the coupling between the control loops, the net effect is that actions at one level cancel or interfere with the actions at another level with a net unsatisfying result.

### 3.3. Sensemaking/muddling through

As noted in the opening of this section, the quality of the overall system performance will depend jointly on its ability to respond effectively to both the observer demands associated with detecting the signals in the noise and the control demands associated with the speed/accuracy constraints for correcting deviations from intentions (i.e. errors). The sensitivity to surprise in updating situation awareness and the sensitivity to error with respect to deciding and acting will determine the system's capability to appropriately satisfy the stakeholder value propositions against which system performance will be assessed (e.g. see Rudolph, Morrison, and Carroll 2009).

The complexity of the joint demands of observing and controlling in complex sociotechnical systems and the implications for effective decisions and action have been well articulated in Lindblom's (1959, 1979) classic papers on ‘muddling through’ and more recently in Weick's (1995) construct of ‘organisational sensemaking’. For example, Lindblom's construct of muddling, where he suggests that effective policy-making in complex organisations involves an incremental approach of trial and error, as opposed to any formally computational approach to making absolutely right decisions, is consistent with the observing and controlling framework suggested here. Or consider Weick's observations about the need to ‘act into’ situations and then make sense of the consequences retrospectively in order to shape future decisions. This fits nicely with the metaphor of an organisation that acts to simultaneously achieve goals (control) and to generate information needed to evaluate beliefs (observe).

In a retrospective assessment of ‘muddling through’ Lindblom (1979) writes:

Perhaps at this stage in the study and practice of policy making the most common view (it has gradually found its way into textbooks) is that indeed no more than small or incremental steps – no more than muddling – is ordinarily possible. But most people, including many policy analysts and policy makers, want to separate the “ought” from the “is” They think we should try to do better. So do I. What remains as an issue, then? It can be clearly put. Many critics of incrementalism believe that doing better usually means turning away from incrementalism. Incrementalists believe that for complex problem solving it usually means practicing incrementalism more skilfully and turning away from it only rarely.

This observation seems to be consistent with the constraints associated with stability of the joint observing/controlling problem. For example, from the point of view of managers changing policy in complex sociotechnical systems, there will typically be long lags between the implementation of a change in policy and the ultimate feedback that results from the percolation of that change through the many nested control loops in the system. In the food industry, the impact of choosing an enzymatic detergent on worker safety will only be apparent in the long run (integrating over many restaurants over long periods). Thus, consistent with the demands for stability, it is prudent to make small changes and to take time to monitor the impact of those changes (i.e. it is prudent to take an incremental approach). However, when managing a crisis, involving lives or money, it may be necessary to take larger actions. As the Millstone case illustrates, even a larger action, such as replacing managers, is but one iteration in a change process that requires feedback and adjustment, i.e. muddling.

While Lindblom's advice seems wise with respect to higher-level policy decisions (e.g. at the level of regulatory agencies and top management), his analysis does not address the many layers of nested control loops in a complex sociotechnical system. Thus, for example, correcting the safety culture in an organisation like Millstone does not depend simply on a single change in management policy, but rather depends on multiple actions implemented at multiple levels. This included replacing vice presidents, hiring new people to manage the employee safety concern programmes, encouraging employees to contribute and take risks by doing the right thing (which formerly got them in trouble with management) and ultimately training line managers to be more receptive (better listeners/observers) and more responsive (higher gain controllers) to the concerns of the staff. Thus, while a low gain, incremental approach may be generally wise with respect to management policy, implementing these changes may require enabling people lower in the organisation to be more responsive (higher gain) to local problems/demands.

The need to distribute authority to lower levels in an organisation is a common observation associated with organisations that have to cope with high risks in dynamic environments. This is consistent with the requirement that for coordination in a complex multi-level sociotechnical system, it is necessary to match the controller's authority with the observer's capability for each control loop. Thus, stability often depends on empowering those with unique access to local information to act on that information, rather than to pass that information up the chain and to wait for authorisation from a higher authority. In military systems, this is recognised in the construct of ‘command intent’ (Shattuck 2000). The idea is that top levels within the organisation set general guidance about the overall goals (e.g. the tactical objectives), and the choices associated with specific actions are left to lower levels (e.g. field officers) who have direct information about the local situations. In these dynamic contexts, formally hierarchical organisations become flatter to behave more like heterarchies or networks when dealing with high tempo risks. That is, authority for action shifts to lower levels in the hierarchy, as a function of shifts in access to information. Thus, for both the Millstone and slipping cases, it will be important to consider whether decisions are being made at the appropriate levels. For example, could franchises advise managers about the tradeoffs associated with enzymatic and non-enzymatic cleaners and let them decide what type of cleaner will work best for a particular restaurant?

There are several properties that seem to differentiate organisations in terms of their ability to cope with the observing/controlling demands in complex environments. First, these organisations often have rich communication networks (e.g. voice loops). These networks can help increase accesses to information (although they can also be a source of noise) relative to the observing problem, to help ensure that the right hand knows what the left hand is doing in order to potentially reduce interference from control actions in multiple loops (e.g. Patterson, Watts-Perotti, and Woods 1999).

Second, high reliability systems often have high levels of mobility across the levels and within levels. For example, this might reflect people starting at the bottom and moving up through levels of the organisation. Or it might reflect a policy of cross-training or job switching so that people have shifting jobs or responsibilities over their careers. This is useful because people have an opportunity to see the problem from many different perspectives (e.g. Rochlin, La Porte, and Roberts 1987). This helps the individual internal models to converge on common ground and to make local actions (both communications and decisions) that take into account the larger context.

Finally, high reliability systems typically have flexible allocation of control authority. This allows the system to self-organise into locally smart units or microsystems, where the people with the most relevant information (i.e. most direct access to relevant feedback) are empowered to make local decisions informed by the global guidance (i.e. command intent) from above. Thus, the system can have high gain locally to respond to dynamic threats, without the risks of instability that can result from long lags in the control loops associated with communications to and from a centralised authority. Of course, if local authority is not coordinated within the overall context (i.e. common ground and global intent), then instability is likely. Again, it is important to emphasise the need for balance between empowering local components to act, on the one hand, and constraining them so that their actions are coordinated with the actions and demands associated with other components, on the other. In dealing with complexity this means finding a stable middle ground between hierarchical and anarchical forms of organisation.

## 4. Discussion and conclusions

## 4.1. Implications for improving coordination in sociotechnical systems

The control theoretic framework suggested here is intended as a guide for improving intuitions about the dynamics of sociotechnical systems, but it is in no way an answer, solution or prescription for success. Also, we are not suggesting the application of analytic control models for building either simulations or models of these systems. Even if building accurate analytic models was possible, the models themselves would be at such a level of complexity that their behaviour would be no less surprising than that of the systems they represented. If there is a value to this abstract framework, it is as a guide for generalising from one sociotechnical system to another, both in terms of potential problems and in terms of potential solutions. It can also be a guide for generalising across research programmes at different scales. For example, it might help us to see parallels from the literature on naturalistic decision-making that are relevant to the literature on organisational sensemaking; or it might help us to see parallels from the literature on skilled motor coordination with the literature on team coordination and distributed work.

In the opening of this paper we made the claim that *Context Matters!* when it comes to specifying the boundaries of a cognitive system, thus motivating a sociotechnical perspective. However, when it comes to practical solutions, we claim that *Details Matter!* The ultimate value of the control theoretic framework is to provide a holistic view to help guide our explorations of the details. As such, it can suggest ways to bound our explorations (i.e. to help us to identify the sociotechnical system), highlight the details that might make patterns more evident and perhaps more sensible, and suggest structures and measures (e.g. delays in feedback loops) that may be particularly relevant to system performance. But this framework provides no easy answers. The biggest potential mistake in applying this framework would be to trivialise the problems of communication and decision-making to fit tractable analytic solutions. Our own experiences with this framework is that it helped us to discover that the problems are often more complex than they first appeared, and definitely more complex than suggested by simple analytic models of observers and control mechanisms.

### 4.2. Implications for practice

Our reframing of communication and decision-making practices in terms of observability and controllability has several important implications for managing safety in complex sociotechnical systems. First, we must be able to ‘see the system’. The frameworks we have presented provide an outline of the multi-level and multi-stakeholder relationships that constitute organisations, whether fast food chains, nuclear power plants or other entities. Only when we recognise that microsystems such as a work shift are embedded in departments, organisations, industries, etc. can we begin to map and understand the communication interfaces and decision points, and then to develop recommendations for improving observability and controllability to meet safety goals. This provides a framework for encouraging managers and policy-makers at multiple levels to engage broadly with the social system, seeking diverse viewpoints, discussing shared values and goals, achieving common ground and developing creative ideas. An effective process might produce not only an accurate and useful set of information, but also contribute towards improving the climate of mutual understanding, respect, trust and collaboration that could enable improvements in both productivity and safety.

In the fast food and Millstone cases, seeing the system and engaging more representatives of the system were and are challenges. Research on slips and falls in the fast food industry is sparse. Only recently are we beginning to understand the full system. Epidemiological research by itself only hints at the complex processes underlying accident rates. Regulators, franchise owners, managers and shift supervisors have very distinct, local understanding of their part of the system. Mandates from above do not necessarily work, as demonstrated by the failure to implement the required cleaning practices. At Millstone, the new chief officer did not understand the sociotechnical system, despite having worked at Millstone early in his career and having years of experience running another nuclear power plant. His understanding grew when he admitted to mistakes and created conditions to engage more people in the change process.

The above discussion introduces the second point, that rich communication channels and venues are needed to share information and interpretations. For a system to coordinate complex operations, please multiple stakeholder groups and adapt to internal and external changes, there must be a great deal of communication in all directions. In order for such communication to occur effectively, the people involved have to have sufficient trust and shared values and goals such that they are willing to speak up, and listen. In addition, they need to actively build common ground so that they can understand concerns associated with components that are distinct from their own local perspective. A well-functioning organisation is full of formal and informal communication opportunities, from regular meetings (whether in person or virtual) to informal networks and gathering places where people come together naturally. Fast food restaurants that have managers only on day shift while important work is going on during night shift must find some compensatory mechanism for increasing observability and controllability. This might include occasional manager visits at night or delegation of tasks to shift supervisors, lead workers, specialised safety roles, or self-managed work teams.

The third point is that in complex systems, decision-making is distributed and dynamic. Decisions relevant to safety are occurring all the time, in different locations in the organisation. In the fast food case, decisions about cleaning strategies are made in one location, hiring decisions are made elsewhere, training materials are developed in yet another place and so on. The fact that in a sociotechnical system the ability of workers to wash floors with enzyme cleaners involves an interaction among these decisions appears to be opaque to many of the store managers and franchise owners. As you change one feature of the floor cleaning process, the consequences reverberate through many interdependent decisions, but the nature of those consequences may be separated in time and space from the decisions themselves. We know that systems with long delays and many interrelated cause–effect relationships are hard to understand and hard to manage (Perrow 1999; Sterman 2000).

A core theme running through the fast food and Millstone cases is the level of trust between workers and managers, or between departments of an organisation. We know that trust strongly affects the flow of information (Mayer, Davis, and Schoorman 1995). Trust also affects the nature of decision-making in terms of who is willing to make a decision, who participates in the decision process, who is willing to implement a decision made by others and so forth. We know that trust is difficult to win, and easy to lose. It would be helpful to understand these relationships in more detail, particularly how levels of complexity, levels of hazard and levels of trust interact. The fast food industry seems to trust the expertise of technical experts in central locations, but not the expertise of workers actually doing the job. This is exacerbated by the high turnover and variable language skills among the lowest level workers. But is this a self-fulfilling prophesy in which mistrust breeds ignorance and resistance? That is what happened for a time at Millstone, where workers automatically assumed that anything management wanted must be bad for the workforce. Over time, management was able to build trust, primarily by actions rather than words.

### 4.3. Implications for research

When we look at the research literature with respect to communication, decision-making and organisational performance, we see an expanding argot of terms to describe the performance dynamics: situation awareness, recognition-primed decision-making, naturalistic decision-making, ecological rationality, situated action, organisational sensemaking, high-reliability organisations, learning organisations, macro-cognition, common ground, embodied cognition, distributed cognition, resilient organisations, metacognition, organisational readiness, etc. On the bright side, this expansion reflects an increasing appreciation for the complex dynamics of sociotechnical systems. However, the potential dark side is when the exploding argot becomes an obstacle to understanding these dynamics (e.g. Flach 2008). We worry that this not only creates confusion and unproductive debates within the specific fields of human factors and cognitive systems engineering, but it also has the potential of isolating these fields from other disciplines that can potentially contribute towards the scientific and practical challenges presented by sociotechnical systems.

We worry that within the field we are losing common ground and we think that a first step towards recapturing common ground would be to reconsider some of the new insights reflected in the expanding argot relative to the foundations of general systems theory. Which of the new terms reflect truly unique phenomena, and which of the new terms simply amplify insights that can be articulated equally well in the language of general systems theory?

The other major impact of a dynamical systems perspective on sociotechnical organisations is that it requires that we change the underlying scientific narrative (e.g. Juarrero 1999). That is, it requires that we change the research focus from narratives based on *causes that determine performance trajectories* to narratives that focus on *constraints that shape fields of possibilities*.

The observer/controller metaphors are important steps for bringing both ‘time’ and ‘context’ back into the theoretical landscape. Today, the emerging field of nonlinear dynamics (e.g. chaos theory) is providing an alternative narrative to the old ‘mechanistic’ view of causality. This has important practical consequences for the basic research agenda.

There is an increased need for naturalistic observations of sociotechnical systems and associated approaches such as work analysis (e.g. Flach, Mulder, and van Paassen 2004; Vicente 1999) to provide insights into the dynamics of work situations. In describing field observations, it will be necessary to shift attention from a search for the ‘root causes’ of a phenomenon to a way to characterise the constraints that shape the flows of information and the possibilities for actions (e.g. Leveson 2012).

In classical paradigms the primary focus was on predicting specific trajectories of behaviour. In the control theoretic paradigm, the focus needs to shift to mapping out the boundaries (e.g. state space representations) that constrain access to information and possibilities for action relative to value systems (e.g. multiple goals and payoff matrices). As with other nonlinear phenomenon (e.g. weather systems), these models will be vulnerable to butterfly effects – such that small local changes may have dramatic impacts on the predictions of behavioural trajectories. Thus, the models will not allow precise predictions about the specific trajectory of any sociotechnical system, but they will support broad generalisations about regions of possibilities. For example, it will be possible to talk in general about properties (e.g. rich information networks) that might contribute to improving the resilience of an organisation for adapting to change. But it may not be possible to ever predict the specific impact of any particular action or decision.

It is important to realise that these limitations with respect to predicting behavioural trajectories are not unique to cognitive or sociotechnical systems and it does not imply that these systems are not lawful. Rather, the limitations of science and predictions reflect actual limits on observability and controllability with respect to the complexity of all natural systems (Mandelbrot 1983). In the same way that weather systems and other nonlinear phenomena test the limits of simple physical models, sociotechnical systems test the limits of simple cognitive and organisation models.

Part of the theoretical challenge will be to map out the space of relations among three dimensions of sociotechnical systems that must be explored: (1) the demands created by different work domains, (2) the types of organisational and social structures and (3) the opportunities afforded by advancing technologies.

#### 4.3.1. Differential demands of work domains

The two cases presented in this paper were chosen to represent very different types of work domains. At one end of the work demand spectrum, there are systems that are tightly constrained by physical processes such as nuclear power and aviation systems. In these domains, there will typically be a steep information gradient from the sharp end of the system, where the operators live, up the chain to management and ultimately to regulatory and political decision-makers. In such systems, operators will typically be in a privileged position with respect to access to information and capability to meet the dynamic control demands relevant to the overall safety of the processes. Higher levels in these organisations will typically impact safety through the selection and training of these operators and by ensuring that these operators have the resources (e.g. information) and authority they need to make smart decisions with regard to directly managing the process.

The cases of slips in the fast food industry and medical errors represent another end of the work domain continuum. In these systems, processes are highly variable (e.g. every restaurant and every patient is slightly different from the others) and highly distributed (e.g. each component at the sharp end has a restricted access to information). Thus, in considering safety relative to situations such as slip accidents and medical errors, it will be difficult to identify problems at the sharp end of the system, because there will be limited samples and many sources of variability (e.g. clumsy individual or unsafe conditions). For these systems, the problems often can only be observed through integration over larger samples than are available to those working at the sharp end of the system. In fact, the people in these systems are often quite surprised to discover the magnitude of the problem when the data are aggregated and examined (e.g. Brennan et al. 1991; Institute of Medicine 1999). As noted earlier, the discovery of the problems with enzymatic cleaners was found in a research programme to examine the potential of slip-resistant shoes for reducing slipping accidents.

#### 4.3.2. Differential types of organisations

A second dimension of the theoretical space requires consideration of the different types of organisational structures. For example, there is a long history of debate over different organisational structures with regard to economic systems (e.g. Hayek 1945). Also, in the military domain, there has been extensive discussion of the relative effectiveness of hierarchical versus network forms of organisation. For example, Arquilla and Ronfeldt (1997) assert that:

… the information revolution favors and strengthens network forms of organisation, while making life difficult for hierarchical forms. (5)

Despite these claims, there are strong arguments that network forms of organisation will not be stable unless they have appropriate information constraints such as that reflected in the constructs of ‘command intent’ and ‘imparting presence’ in the military domain (Shattuck 2000). These constructs emphasise that discretion for junior officers in a military organisation to take an initiative to adapt to local constraints must be constrained by a common ground with regard to the global mission.

Observations of high reliability systems (e.g. Rochlin, La Porte, and Roberts 1987) suggest that there may not be a single stable organisational structure. Rather, these systems appear to ‘self-organise’ to meet the changing demands of hazardous work domains. In self-organising systems, there may be a heterarchical form of control, where there is a changing locus of centralised control that shifts as a function of access to the information and resources needed to keep pace with rapidly changing processes.

Sage and Cuppan (2001) describe another organisational structure that seems to be emerging as a strategy for coping with complex domains such as emergency operations in response to large-scale disasters (e.g. Flach et al., 2014). This is the ‘Federation of Systems’. This is described as a specific class of system-of-systems where multiple component systems collaborate, yet there is ‘little central power or authority for command and control’ (327). These components (e.g. hospitals, police, fire and relief organisations at state and federal levels) join in a coalition to meet the common needs of the federation (e.g. to respond to a regional disaster).

These are just a few examples that reflect different types of organisations, with some indication of the differential demands on observability and controllability. While there are many claims about the benefits and demands of alternative forms of organisation, there is a need for a global theoretical framework to differentiate across organisation types and to specify the implications of specific forms of organisations with regard to communication and control.

#### 4.3.3. Technological opportunities

It seems apparent that the evolution of sociotechnical systems and the associated interests of both scientists and practitioners are being driven in part by the opportunities afforded by emerging information technologies. These technologies are transforming both the nature of work and the nature of organisations. In terms of work, there is a trend for increased distance between workers and the products of work. As Rochlin (1997) observes, in the industrial age, work shifted from direct production of goods and services to the operation of production machinery; in the information age work is shifting further away from production, such that workers are now often managing computers that control the production machinery. In terms of organisations, information technologies are now allowing information and control to be distributed in ways that allow real-time collaborations across distances that would have been unthinkable a generation ago.

It should be clear that while information technologies support new opportunities for collaboration across space and time, these technologies do not determine the forms of the organisations. However, in the absence of a theoretical framework, the opportunities afforded by these technologies for short-term increased efficiencies may lead to the design of brittle systems that undermine overall safety or long-term stability. In particular, Rochlin (1997) highlighted the need to differentiate between ‘waste or slop’ versus ‘slack’ or ‘necessary friction’. Slack and necessary friction refer to the buffering capacity that allows organisations and social systems to maintain stability in the face of unanticipated variability. In essence, Rochlin is highlighting the balancing act that all control systems must address – between being too conservative (sluggishness or waste) or too aggressive (e.g. over control leading to pilot induce oscillations) in adjusting to error or changing situation demands.

## 5. Conclusion

In sum, the challenge of safety in sociotechnical systems requires us to consider the opportunities for improving the interactions between the structure of organisations, the demands of work ecologies and the technologies for communication and decision-making. We suggest that it might be productive to frame this challenge in control theoretic terms in relation to differential demands for observability and controllability and the implications for stability. Observability reflects issues of communication to ensure that each control component has the information (feedback) needed for both local stability with respect to the processes directly under control and global stability with respect to coordination with control components at higher and lower levels within the sociotechnical system. Controllability reflects issues of decision-making that determine whether the authority to act is commensurate with the available information and the dynamic demands of the work processes (e.g. in terms of time constants and windows of opportunity).

This need for a control theoretic approach to safety in sociotechnical systems has been previously articulated by Rasmussen and Svedung (2000), and more recently by Leveson (2012). However, the need to balance the demands on control and observation has long been appreciated by organisational theorists. As March (1991) observed, control (exploitation) and observation (exploration) go hand in hand in managing complexity:

A central concern of studies of adaptive processes is the relation between the exploration of new possibilities and the exploitation of old certainties …. Exploration includes things captured by terms such as search, variation, risk taking, experimentation, play, flexibility, discovery, innovation. Exploitation includes such things as refinement, choice, production, efficiency, selection, implementation, execution. Adaptive systems that engage in exploration to the exclusion of exploitation are likely to find that they suffer the costs of experimentation without gaining many of its benefits. They exhibit too many undeveloped new ideas and too little distinctive competence. Conversely, systems that engage in exploitation to the exclusion of exploration are likely to find themselves trapped in suboptimal stable equilibria. As a result, maintaining an appropriate balance between exploration and exploitation is a primary factor in system survival and prosperity. (71)

## Footnotes

^4.^ All quotations pertaining to the Millstone case are taken from this article.

^5.^ All information relative to slips in the limited food service injuries is taken from these sources.
